# Fulminant elderly adult-onset Still disease effectively treated with tocilizumab and methotrexate: A case report

**DOI:** 10.1097/MD.0000000000029354

**Published:** 2022-07-15

**Authors:** Yugo Horiuchi, Kenichi Hashimoto, Hideyuki Horikoshi, Azusa Sano, Yusuke Kawamura, Naoya Fujita, Motohiro Kimata, Yosuke Ono, Yasuhiro Obuchi, Arisa Makino, Mayuko Kaneko, Fumihiko Kimura, Kenji Itoh, Yuji Tanaka

**Affiliations:** a Department of General Medicine, National Defense Medical College, Tokorozawa, Saitama; b Division of Hematology and Rheumatology, Department of Internal Medicine, National Defense Medical College, Tokorozawa, Saitama; c Department of Integrative Physiology and Bio-Nano Medicine, National Defense Medical College, Tokorozawa, Saitama; d Department of Traumatology and Critical Care Medicine, National Defense Medical College, Tokorozawa, Saitama.

**Keywords:** adult-onset Still disease, elderly, interleukin-18, macrophage activating syndrome, tocilizumab

## Abstract

**Rationale:**

Adult-onset Still disease (AOSD) is a rare inflammatory disease of unknown etiology. AOSD is common in young or middle-aged adults; however, in recent years, there have been increasing reports of elderly AOSD. Differentiating AOSD from diseases such as infections and malignancies is difficult. Moreover, rare fulminant AOSD cases with resistance to corticosteroids and immunosuppressive drugs have been reported.

**Patient concerns:**

An 80-year-old woman presented with flaccid fever, generalized arthralgia, and erythema of the anterior chest for 2 weeks. On day 5 of hospitalization, the patient developed pleural effusion with hypoxemia and her vital signs indicated rapid progression to shock. During the clinical course, the levels of inflammatory markers, including maximum level of ferritin and white blood cells (WBCs) were elevated (252,796 ng/mL and 86,500/μV, respectively) with disseminated intravascular coagulation syndrome (DIC) and macrophage activation syndrome (MAS).

**Diagnosis:**

The patient was diagnosed with elderly AOSD as per the Yamaguchi criteria for AOSD. The state of disease was extremely severe with rapid progression and was, thus, categorized as a fulminant form of elderly AOSD.

**Interventions:**

The patient was treated with prednisolone (PSL) pulse therapy (1000 mg/d) twice and plasma exchange in the intensive care unit for the primary disease and shock. Although she recovered from shock, she developed DIC and MAS. Methotrexate (MTX; 10 mg/d) improved the DIC and MAS. However, severe pleuritis recurred and the patient developed pericarditis; her primary disease was poorly controlled. Finally, tocilizumab (TCZ) was introduced using interleukin-18 (IL-18) as a surrogate marker. The IL-18 level was measured repeatedly following admission, with the peak level (170,000 pg/mL) recorded on the 75th day of hospitalization, immediately prior to introducing TCZ.

**Outcomes:**

The combined use of MTX, TCZ, and PSL was effective in suppressing elderly AOSD, which was unsuccessfully controlled with MTX and PSL. Frequent monitoring of IL-18 levels proved useful for differentiating elderly AOSD from other diseases.

**Lessons:**

A fulminant form of elderly AOSD was treated with a combination of MTX, TCZ, and PSL. Repeated monitoring of IL-18 levels can be useful for decision-making in treating elderly AOSD.

## 1. Introduction

Adult-onset Still disease (AOSD) is a relatively rare systemic inflammatory disease that presents with various symptoms, including fever, joint pain, and skin rashes.^[1]^ The incidence of AOSD in elderly patients is lower than that in young or middle-aged individuals.^[1]^ However, recently, an increasing number of studies have reported an increase in the prevalence of elderly AOSD.^[2]^ Although prednisone (PSL) and immunosuppressive agents are recommended as standard therapies for AOSD, these drugs are ineffective in certain severe cases. AOSD is rarely associated with fatal complications, such as disseminated intravascular coagulation syndrome (DIC) and macrophage activation syndrome (MAS).^[2]^ In addition, in rare instances, it can be complicated by a fulminant form of myocarditis, which can progress rapidly.^[3]^

Interleukin-18 (IL-18) levels are strongly associated with AOSD.^[4–9]^ In fact, IL-18 measurement is useful for differentiating AOSD from other diseases, particularly infections, and collagen disorders, as well as in determining disease severity and treatment response.^[4–8]^ Although some case reports suggest that IL-18 measurement is useful for assessing AOSD severity,^[9]^ there are few reports on the use of repeated IL-18 measurements throughout the full management course of AOSD as a guide for differential diagnosis and treatment.

Herein, we report the case of a patient who presented with a fulminant form of elderly AOSD accompanied by DIC and MAS. Combination therapy of tocilizumab (TCZ), methotrexate (MTX), and prednisolone (PSL), guided by repeated monitoring of IL-18 levels, was effective in suppressing the condition.

## 2. Case report

**Figure 1.**
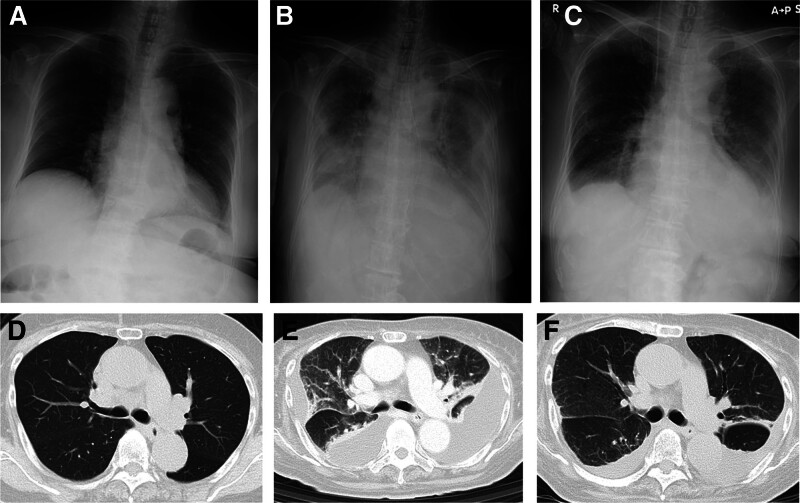
Chest X-P (1A) and chest CT (1D) on admission (day 1) showing no interstitial shadows or pleural effusion. Chest X-P (1B) and chest CT (1E) on day 70 after admission and before introduction of TCZ. Significant interstitial shadows and pleural effusion can be seen. At this point, the patient underwent weekly thoracic drainage due to increased pleural effusion over time and repeated hypoxemia. Chest X-P (1C) and chest CT (1F) post-TCZ induction (day 100) (chest X-P after day 2 of initiating TCZ). Interstitial shadows and pleural effusion are improved. CT = computed tomography, TCZ = tocilizumab.

An 80-year-old Japanese woman was referred to our institution complaining of a spiking fever and joint pain. One week before admission, she had a fever of over 39°C, accompanied by sore throat and joint pain. At the time of admission, swelling with tenderness of the right hand, both knee joints, and a red skin rash on the anterior chest were observed. However, interstitial pneumonia and pleural effusion were not evident at that time (Fig. 1A and D). Laboratory tests on admission showed elevated levels of serum ferritin (39,000 ng/mL), leukocytes (16,900/µL), C-reactive protein (CRP; 23 mg-dL), IL-6 (470 pg/mL; reference value < 4 pg/mL), and IL-18 (74,300 pg/mL; reference value < 211 pg/mL). The antinuclear antibody and rheumatoid factor test results were negative and a random skin biopsy showed no malignant tissue nor malignant lymphoma. After excluding other collagen diseases, infection and malignancy, a diagnosis of elderly AOSD was made according to Yamaguchi classification criteria.^[10]^

On day 5 of hospitalization, the patient had a high fever ranging between 39°C and 40°C, her systolic blood pressure dropped to the 70 to 90 mm Hg range with a disturbance in consciousness, and hypoxemia due to pleural effusion. Since white blood cell (WBC), ferritin, and IL-18 levels were elevated, a cytokine storm with shock due to increasing elderly AOSD severity was highly suspected. Consequently, intravenous methylprednisolone pulse therapy (1000 mg/d) for 3 days was administered resulting in temporary improvement in consciousness and vital signs. However, on day 9 of hospitalization, the patient again developed a high fever (> 40 °C) and shock vitality. Thus, we readministered methylprednisolone pulse therapy using the same protocol; however, this course of treatment was unsuccessful at managing elderly AOSD. Due to hypoxemia caused by pleural effusion, resulting from pleuritis, oxygen was administered using 10 L/min reservoir masks. From day 14 of hospitalization, plasma exchange was initiated with noradrenaline perfusion in the intensive care unit. Plasma exchange was performed a total of 4 times, resulting in a temporary decrease in pleural effusion. She recovered from shock and hypoxemia. In addition, there was a slight improvement in the levels of inflammatory markers, including WBC, ferritin, and IL-18.

**Figure 2.**
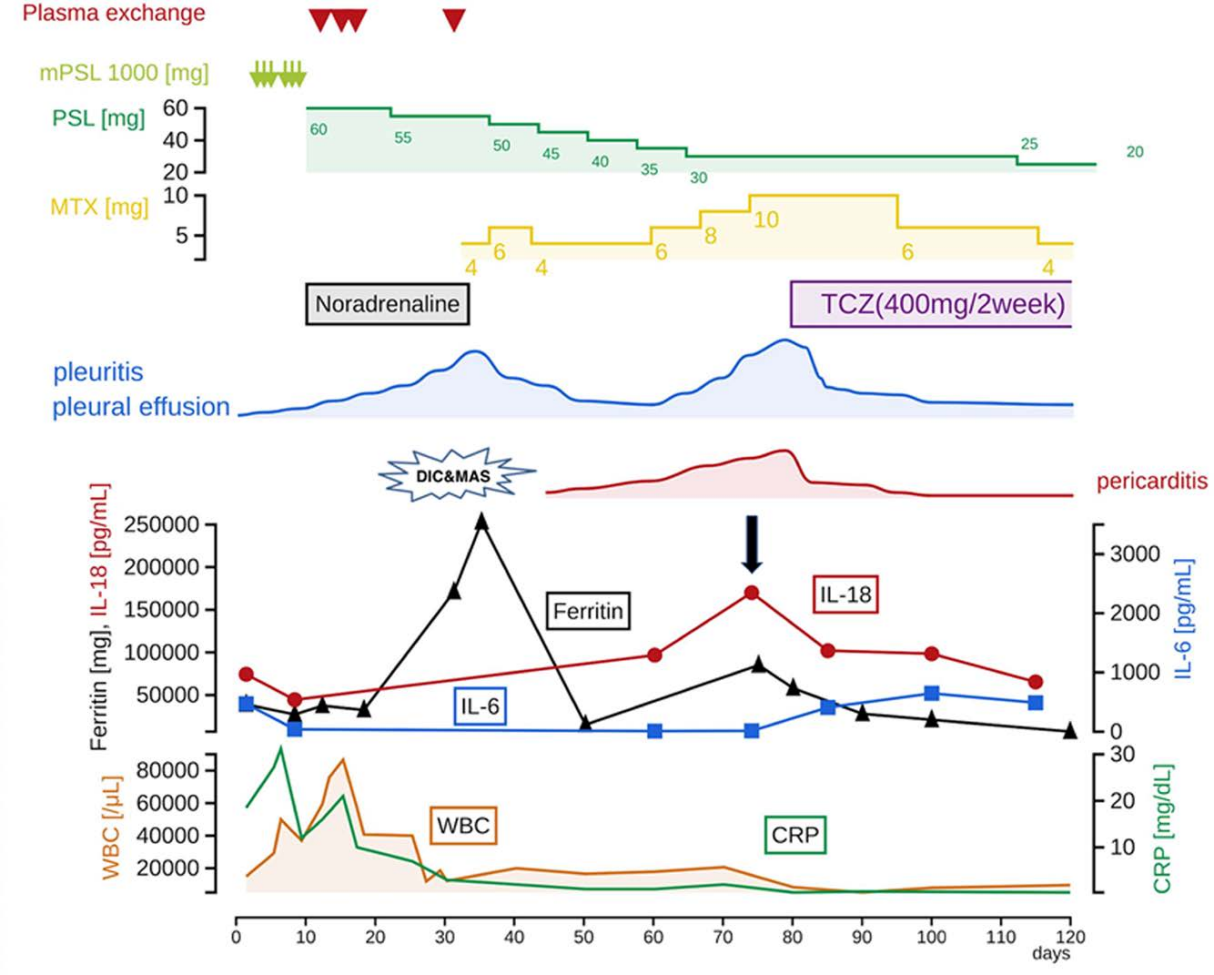
Clinical course. CRP = C-reactive protein, DIC = disseminated intravascular coagulation, IL-18 = interleukin-18, IL-6 = interleukin 6, MAS = macrophage activation syndrome, mPSL = methylprednisolone, PSL = prednisolone, TCZ = tocilizumab, WBC = white blood cell.

**Figure 3.**
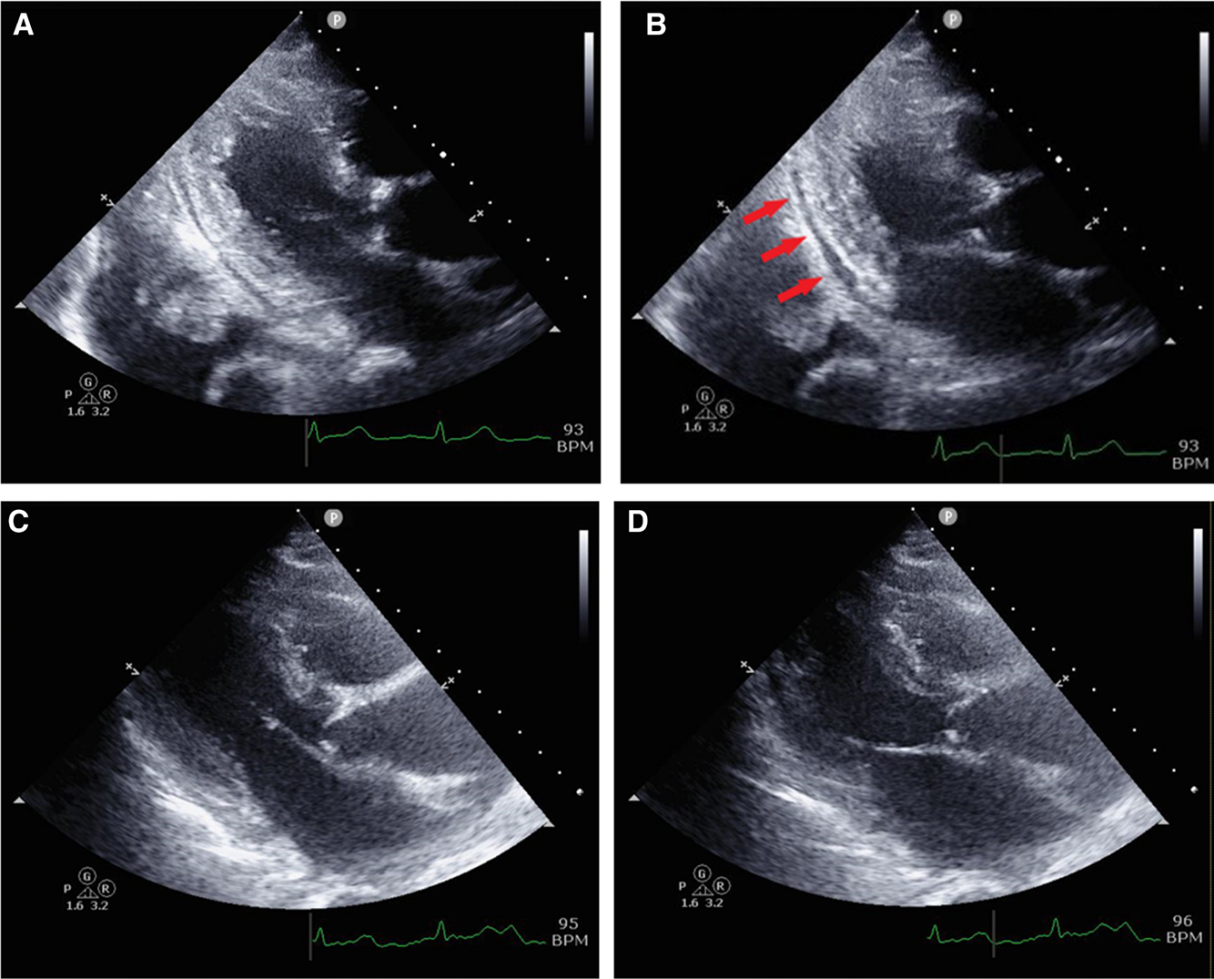
Pericardial effusion of approximately 1 cm was observed in the posterior wall of the left ventricle, and complications of pericarditis due to increased severity of elderly AOSD were suspected (A, diastole; B, systole). Pericardial effusion was improved post-TCZ induction (day 100) (C, diastole; D, systole). AOSD = adult-onset Still disease, TCZ = tocilizumab.

However, on day 31 of hospitalization, anemia (Hb 6.1 g/dL) and thrombocytopenia (Plt 12,000/µL) progressed with increased levels of WBC and ferritin of 86,700/µL (day 15) and 252,796 ng/mL (day 36), respectively. Her blood test results met the Japanese Acute DIC diagnostic criteria.^[11]^ Furthermore, a bone marrow biopsy showed findings relevant to hemophagocytic syndrome (HPS) and met the autoimmune-associated HPS diagnostic criteria for MAS.^[12]^ On day 33 of admission, along with coping strategies for DIC and MAS, oral administration of MTX was started at 4 mg/week for elderly AOSD. She recovered from DIC and MAS; however, after increasing the dose of MTX up to 10 mg/wk, pleural effusion was not fully controlled. On day 70 of admission, interstitial shadows and worsening pleural effusion with hypoxemia were observed with increased severity (Fig. 1B and E). The patient required weekly thoracic drainage due to increased pleural effusion over time and repeated hypoxemia. Although WBC and CRP levels did not increase, ferritin levels increased from 15,230 to 85,000 ng/mL, and IL-18 increased from 96,800 to 170,000 pg/mL (day 75; Fig. 2, black arrow). Meanwhile, the concentration of IL-6 was 12.6 pg/mL (IL-18/IL-6 ratio, 170,000:12.6 [13,492.1]). Pericardial effusion due to pericarditis was observed (Fig. 3A and B) with uncontrolled fever and anemia. Therefore, a relapse of elderly AOSD was suspected; consequently, intravenous administration of TCZ 400 mg was initiated for 2 weeks (Fig. 2). Following administration of TCZ, the pleural effusion and interstitial shadowing improved rapidly (Figs. 1C and F and 2). In addition, pericardial effusion was significantly reduced (Fig. 3C and D), and inflammatory markers, including WBC, ferritin, and IL-18, exhibited monotonic decrease (Fig. 2). Four months after hospitalization, the patient did not exhibit any adverse events from TCZ and her clinical symptoms improved. Subsequent PSL and MTX doses were tapered, and ferritin and IL-18 levels decreased 120 days after admission.

## 3. Discussion

Herein, we report the case of a female patient with a fulminant form of elderly AOSD complicated by DIC and MAS, which was extremely severe and progressed rapidly. Elderly AOSD was achieved following the combined administration of TCZ with MTX and PSL. Repeated monitoring of serum IL-18 levels was useful in distinguishing elderly AOSD from other disorders, including infectious and collagen diseases. Moreover, this strategy provided us with practical insights to facilitate future decision-making regarding treatment, particularly in cases that require administration of TCZ.

AOSD primarily occurs in young or middle-aged individuals. The mean onset age of typical AOSD is reportedly between 34 and 36 years,^[13]^ with twin peaks of onset age, namely 15 to 25 and 36 to 46 years also reported.^[14]^ In large epidemiological studies, the age of AOSD onset was reported as 34 ± 23 years (N = 82),^[15]^ 35.2 ± 13.2 years (N = 72),^[16]^ and 37.5 ± 14.8 years (N = 61).^[17]^ In an AOSD epidemiological study, reported by Yamaguchi and Ohta^[18]^ in 1992, the maximum age of developing AOSD was 65 years, with no reported cases older than 66 years. In this study, we therefore defined elderly AOSD as AOSD developing in patients aged > 65 years. Subsequently, we searched PubMed for reports on patients with elderly AOSD and available clinical information, including laboratory data, treatments, and clinical course. To date, 40 cases of patients with elderly AOSD over 65 years of age, including our case, have been reported.^[19–56]^ The clinical and laboratory characteristics of the 40 patients are shown in Table 1. Only 5 of 40 cases were reported between 1991 and 2000, 8 between 2001 and 2010, and the remaining 25 between 2011 and 2020 (Table 1). Notably, most reports of *elderly AOSD* have been reported in Japan (26/40 cases), with the patients being of Japanese descent (Table 1; Table 1, Supplemental Digital Content, http://links.lww.com/MD/G881). The pathogenic mechanism underlying AOSD, and the reason for its increased prevalence in Japanese individuals, remains unclear. However, a potential genetic etiology for AOSD is proposed.^[57]^ HLA DQ B1* 0602 (DQ1) is commonly identified in Japanese patients with AOSD.^[58]^ In addition, the IL-18 S01 haplotype is more prevalent in patients with AOSD than in healthy individuals.^[59]^ Therefore, these genetic factors may contribute to the high incidence of elderly AOSD in Asians, particularly in those of Japanese descent. However, with the increasing number of elderly AOSD cases being reported, it is important to consider it in the proposed differential diagnosis when elderly patients present with unknown fever.

**Table 1:** Characteristics of patients (aged > 65 y) with elderly onset AOSD.

No	Ref No	Age (y), sex	IL-18 (pg/mL)^a^	WBC (/µL)^b^	CRP (mg-dL)^c^	Ferritin (ng/mL)^d^	AOSD severity score† (total) Ref 57	Treatment	Outcome	Published year	Ethnicity
1	^[19]^	83, F	N/A	17,000	5	Over 3000	1	PSL, Aspirin	Remission	1992	Asian (Japanese)
2	^[20]^	72, F	N/A	18,000	16	38,500	1	PSL	Remission	1992	Asian (Japanese)
3	^[21]^	74, F	N/A	31,100	15.36	1,212	2	mPSL pulse, CPA	Remission	1993	Asian (Japanese)
4	^[22]^	74, F	N/A	9,900	10	1,794	3	PSL	Remission	1995	Asian (Japanese)
5	^[23]^	80, F	N/A	15,200	14.5	709	0	Naproxen (NSAIDs)	Remission	1998	Asian (Chinese)
6	^[24]^	74, F	N/A	20,000	N/A	892	3	mPSL pulse, γ-globulin	Death	2002	Caucasian (USA)
7	^[25]^	67, F	N/A	17,000	10.8	18,525	3	PSL	Remission	2002	Asian (Japanese)
8	^[26]^	80, F	N/A	30,000	15	80,000	2	PSL	Remission	2004	French
9	^[27]^	75, M	N/A	17,500	N/A	18,000	2	PSL, MTX	Remission	2004	Hispanic
10	^[28]^	84, F	N/A	18,800	21	44,000	2	mPSL pulse	Death	2005	Asian (Japanese)
11	^[29]^	80, F	N/A	30,700	27.8	21,250	2	PSL	Remission	2005	Asian (Japanese)
12	^[30]^	83, F	125,000	43,800	18.7	10,340	2	mPSL pulse, MTX	Remission	2007	Asian (Japanese)
13	^[31]^	77, F	N/A	17,300	15.1	4,222	3	PSL, plasma exchange	Remission	2007	Asian (Japanese)
14	^[32]^	72, M	N/A	34,200	N/A	2,368	2	PSL	Remission	2011	Caucasian (USA)
15	^[33]^	78, M	N/A	13,000	19.45	2,181	1	PSL, MTX, TAC, TCZ, Etanercept	Remission	2012	Asian (Japanese)
16	^[34]^	83, F	N/A	17,000	38.2	2,000	0	PSL	Remission	2012	Türklers
17	^[35]^	84, M	N/A	18,200	15	4,500	3	PSL, CyA, MTX, Etanercept	Remission	2014	Asian (Japanese)
18	^[36]^	71, F	229,126	5,400	7.5	24,337	6	Half dose of mPSL pulse	Remission	2014	Asian (Japanese)
19	^[37]^	80, F	N/A	9,650	6.5	8,008	1	PSL	Remission	2014	Asian (Japanese)
20	^[38]^	81, F	384,430	24,010	15.31	12,944	4	mPSL pulse, MTX, TAC, CAM	Remission	2015	Asian (Japanese)
21	^[39]^	71, F	N/A	11,520	15.2	107,490	9*	mPSL pulse, TCZ, (TAC)	Remission	2016	Asian (Japanese)
22	^[40]^	88, F	N/A	13,700	20	78,662	8	Half dose of PSL pulse, CyA	Remission	2017	Asian (Japanese)
23	^[41]^	71, F	N/A	35,180	9.97	270	2	PSL	Remission	2017	Asian (Chinese)
24	^[42]^	66, F	140,373	10,500	9	10,000	2	PSL, CyA	Remission	2018	Asian (Japanese)
25	^[43]^	67, F	N/A	12,000	20	N/A	1	mPSL pulse	Remission	2018	Sri Lankan
26	^[44]^	75, F	N/A	6,600	N/A	167,998	7	PSL, PE, CyA, TCZ	Remission	2018	Asian (Japanese)
27	^[44]^	77, F	N/A	11,400	N/A	43,865	6	PSL, PE, CyA	Death	2018	Asian (Japanese)
28	^[45]^	65, F	1,250,000*	16,100	17	3,380	1	PSL pulse, AZP	Remission	2018	Asian (Japanese)
29	^[46]^	74, M	N/A	N/A	N/A	N/A	0	PSL	Remission	2018	Asian (Japanese)
30	^[47]^	65, F	N/A	N/A	14.5	1700	1	PSL, MTX	Remission	2018	Asian (Chinese)
31	^[48]^	69, F	N/A	17,600	44*	3,000	2	PSL, MTX	Remission	2019	African (Tunisia)
32	^[49]^	67, F	N/A	13,760	9.78	3,894	4	mPSL pulse, MTX, TAC, CyA, IVIg, TCZ	Remission	2019	Asian (Japanese)
33	^[50]^	74, F	210,000	20,240	15.7	34,160	5	mPSL pulse, CyA	Remission	2019	Asian (Japanese)
34	^[51]^	82, F	N/A	17,110	28.5	9,899	4	mPSL pulse, CyA, TCZ,	Death	2019	Asian (Japanese)
35	^[52]^	67, F	N/A	18,800	20.1	4,978	2	PSL	Remission	2019	Caucasian (USA)
36	^[53]^	88*, F	43,500	27,800	28	9,619	2	PSL	Remission	2020	Asian (Japanese)
37	^[54]^	77, F	N/A	14,490	7.36	1,500	2	PSL	Remission	2020	Asian (Chinese)
38	^[55]^	73, F	N/A	20,600	19	23,096	5	PSL, anakinra	Remission	2021	Caucasian (USA)
39	^[56]^	84, F	5,000	22,000	30.2	19,491	5	mPSL pulse, CyA, AZA	Remission	2021	Asian (Japanese)
40 (Our case)		80, F	170,000	86,500*	32	252,796*	9*	mPSL pulse, CyA, MTX, TCZ	Remission	2021	Asian (Japanese)

AOSD = adult-onset still disease, AZA = azathioprine, CAM = clarithromycin, CyA = cyclosporine, DIC = disseminated intravascular coagulation, F = female, IVIg = Intravenous immunoglobulin, IL-18 = interleukin-18, M = male, MAS = macrophage activation syndrome, mPSL pulse = methyl prednisolone pulse, MTX = Methotrexate, N/A = Not available, NASIDs = nonsteroidal antiinflammatory drugs, PE = plasma exchange, PSL = prednisolone, ref = reference, TAC = tacrolimus, TCZ = tocilizumab, USA = United State of America.

^*^Maximum value in each category.

^†^AOSD severity score indicated in Supplemental Table 2 http://links.lww.com/MD/G881.

^a^

^b^

^c^

^d^Normal range reference values for each parameter: IL-18 < 211 pg/mL, WBC 3,300 –8,600/μL, CRP < 0.3 mg/dL, Ferritin 23–250 ng/mL, respectively.

Compared to previous reports, the severity of elderly AOSD was the highest in our case, both quantitatively and qualitatively. In quantitative terms, levels of WBC (86,700/µL) and ferritin (252,796 ng/mL), as well as those of other inflammatory markers, were the highest (Table 1). From a qualitative perspective, we evaluated 40 patients according to the AOSD severity score of evidence-based clinical practice guidelines for adult Still disease,^[57]^ which is widely used in Japan (Table 2, Supplemental Digital Content, http://links.lww.com/MD/G881). Only 2 of 40 patients, including the present patient, had 9 of 9 full scores, while the remaining 38 cases were scored ≤ 8/9. Thus, our case involved a patient with extremely severe elderly AOSD, that was rapidly progressing. Suda et al^[50]^ reported that the frequency of DIC was higher in EOSD elderly AOSD than in AOSD, which was also observed in our patient. Furthermore, Tanaka et al^[56]^ compared 22 patients with AOSD (mean age: 35.6 ± 12.8 years) to 16 with elderly AOSD (mean age: 74.4 ± 7.3 years), and reported that the latter group generally exhibited higher MAS incidence, degree of anemia, and thrombocytopenia, which is also consistent with our findings.

The patient in the current study achieved remission after combined administration of TCZ with MTX and PSL, after which elderly AOSD could not be managed with only MTX and corticosteroid therapy. Recently, an increasing number of reports have suggested that TCZ is effective in treating AOSD.^[60–62]^ However, there are no guideline recommendations for the use of TCZ; therefore, its early introduction should be carefully considered. Previously, 4 of 39 patients with elderly AOSD were reportedly administered TCZ (patient numbers 21, 26, 32, and 34; Table 1). In all 4 patients, multiple drugs, including corticosteroids and MTX, were refractory to elderly AOSD. Three of the 4 patients achieved remission, while 1 died due to cytomegalovirus pneumonia. In addition, one of the 4 patients was unable to continue TCZ therapy due to bacterial pneumonia complications, and was switched to etanercept, which achieved remission. Herein, we report the first case of elderly AOSD remission by the combined use of TCZ with MTX and PSL. Current guidelines recommend MTX as a therapeutic agent for steroid resistant or refractory AOSD.^[57]^ Thus, combination of TCZ and MTX has the potential to become an effective treatment strategy for patients with severe elderly AOSD. Meanwhile, Shimizu et al^[63]^ and Kobayashi et al^[64]^ reported that the introduction of TCZ has a risk of inducing fatal complications, such as DIC and MAS. As such, the timing of TCZ introduction should be carefully considered on a case-by-case basis in clinical settings.

In the present case, IL-18 levels were repeatedly monitored from the time of initial diagnosis until remission to differentiate the disease from other conditions, determine treatment efficacy, and evaluate disease severity. Notably, WBC and CRP levels were not elevated during the recurrence of elderly AOSD at day 75, and these inflammatory markers were not sufficient surrogate markers. As elderly AOSD has no specific symptoms or surrogate markers, it is often difficult to differentiate it from other diseases. In particular, in this patient, it was difficult to distinguish elderly AOSD from severe infections and other collagen diseases. Meanwhile, IL-18 levels are a useful marker for differentiating elderly AOSD from other diseases. Indeed, Priori et al^[4]^ reported an IL-18 level of 350 pg/mL as the cutoff for differentiation of severe sepsis from AOSD. If serum IL-18 levels ˃ 1000 pg/mL, AOSD is highly suspected^[7,8]^; however, if it is ˂1000 pg/mL other systemic rheumatic diseases, such as rheumatoid arthritis, systemic lupus erythematosus, Sjögren syndrome, dermatomyositis/polymyositis, or systemic sclerosis should be suspected.^[7,8]^ In our case, IL-18 was 170,000 pg/mL; thus, we strongly suspected the presence of elderly AOSD rather than rheumatic diseases or infection. Accordingly, when the clinical symptoms worsened and IL-18 levels were reevaluated on day 75 (Fig. 3, arrow), we diagnosed the patient with elderly AOSD relapse. To the best of our knowledge, although previous studies have reported IL-18 measurement at the time of initial diagnosis and after treatment, few have reported repeated measurements for follow-up on the prognosis of the disease.

In this case, IL-6 concentration was not a useful marker for therapeutic intervention as it did not exhibit corresponding dynamic changes with disease activity or the levels of IL-18. IL-6 is a cytokine with various biological activities, including inducing the proliferation of T cells, keratinocytes, B cells, and cytotoxic T cells.^[65]^ In addition, it is a key cytokine in rheumatoid arthritis, which is a quasi-syndromic disease of AOSD. AOSD includes systemic and juvenile idiopathic arthritis (JIA), which primarily presents with joint symptoms in children. IL-18: IL-6 in AOSD and JIA indicates that when IL-18: IL-6 > 5000, the probability of AOSD is 100% (11/11); however, when it is <5000, the probability of AOSD is 60% (9/16).^[66]^ In this case, the IL-18: IL-6 was 170,000:12.6 (13,492.1); therefore, it was speculated to be systemic AOSD. Meanwhile, in juvenile rheumatoid arthritis, another quasi-syndromic disease of AOSD, administration of PSL and MTX reduced IL-6 levels to below that of healthy controls.^[67]^ PSL and MTX effectively suppressed IL-6 levels in this case (hospitalization days 8 to 75). Similarly, administration of PSL and MTX could be responsible for the lack of increase in CRP by the mid of the clinical course.

Certain limitations were noted in the current case study. First, in the 39 case reports included, not all data pertaining to blood samples and clinical characteristics of AOSD severity scores were available for the parameters with the highest values (WBC, CRP, ferritin, and IL-18), as well as the clinical characteristics related to AOSD severity score (Table 2, Supplemental Digital Content, http://links.lww.com/MD/G881) listed in Table 1. Additionally, certain case reports lacked various pieces of data (e.g., CRP and ferritin levels). Therefore, disease severity may have been underestimated in some of the cases. Second, we could not determine the IL-18 levels from days 20 to 40 after admission as the patients were transferred to the intensive care unit due to worsening general condition. Therefore, during the period that the patient had developed DIC and MAS, the IL-18 levels may have exceeded 170,000 pg/mL. The blood IL-6 levels increased after the initiation of TCZ therapy (Fig. 2). This phenomenon could be attributed to the blocking of the IL-6 receptors by TCZ. The excess IL-6 can no longer bind to the IL-6 receptors; therefore, resulting in suppressed consumption and subsequently, IL-6 accumulation in the blood.^[68,69]^

## 4. Conclusions

Here, we present a clinical case of a patient with a fulminant form of elderly AOSD complicated by DIC and MAS. The patient achieved remission following combined administration of TCZ with MTX and PSL. Moreover, repeated monitoring of serum IL-18 levels proved useful for informing decisions regarding treatment, in particular regarding the administration of TCZ. Over the years, the incidence of elderly AOSD has increased, and clinicians may encounter patients with severe elderly AOSD in the future. In such cases, TCZ should be considered as a potential treatment option. Moreover, repeated measurement of IL-18 can be a useful strategy for decision-making in treatment selection for elderly AOSD.

### Author contributions

All authors participated in the treatment of the patient and drafted the manuscript. All authors read and approved the final version of the manuscript.

Conceptualization: K.H., H.H., Y.K., K.I., Y.T.

Data curation: Y.H., K.H.

Formal analysis: Y.H., K.H.

Funding acquisition: K.H.

Investigation: Y.H., A.S., K.H., H.H., M.K., A.M.

Resources: K.H.

Software: Y.H., K.H.

Supervision: K.H., Y.T.

Validation: Y.H., K.H.

Writing – original draft: Y.H., K.H., Y.K.

Writing – review & editing: K.H., A.S., H.H., N.F., M.K., Y.O., Y.O., M.K., A.M., F.K., K.I., and Y.T.

## Supplementary Material


