# Supplementary material for: Fulminant elderly adult-onset Still disease effectively treated with tocilizumab and methotrexate: A case report
Source: Medicine (Baltimore). 2022 Jul 15;101(28):e29354. doi: 10.1097/MD.0000000000029354 (PMC11132312; doi:10.1097/MD.0000000000029354)
Supplement: Supplementary file 1 [file medi-101-e29354-s001.pdf]

Supplementary Table 1

Number of published EOSD case reports by race globally

Asian (Japanese)	27
Asian (Chinese)	4
Caucasian (USA)	3
African (Tunisia)	1
Turkish	1
French	1
Hispanic	1
Sri Lankan	1
**Total**	**40**

Supplementary Table 2

AOSD severity scoring[57])

Serositis	None 0	Yes 1
DIC	None 0	Yes 2
HPS	None 0	Yes 2
Neutrophilia	None 0	Yes 1
High levels of ferritin	None 0	Yes 1
Lymphadenopathy	None 0	Yes 1
Steroid resistance	None 0	Yes 1
**Total Score**		**0–9 points**
